# Case Report: First Case of Non-restrictive Ventricular Septal Defect With Congestive Heart Failure in a Chinese Han Male Infant Carrying a Class II Chromosome 17p13.3 Microduplication

**DOI:** 10.3389/fped.2022.825298

**Published:** 2022-02-28

**Authors:** Yung-Yu Yang, Chun-Ting Liu, Li-Fan Pai, Chih-Fen Hu, Shyi-Jou Chen, Wan-Fu Hsu

**Affiliations:** ^1^Department of General Medicine, Tri-Service General Hospital, National Defense Medical Center, Taipei, Taiwan; ^2^Department of Pediatrics, Tri-Service General Hospital, National Defense Medical Center, Taipei, Taiwan; ^3^Graduate Institute of Microbiology and Immunology, National Defense Medical Center, Taipei, Taiwan

**Keywords:** congenital heart disease, congestive heart failure, chromosome 17p13.3 microduplication, microarray comparative genomic hybridization, ventricular septal defect

## Abstract

Chromosome 17p13.3 microduplication syndrome is considered a multisystem disorder that results in a wide variety of clinical manifestations including dysmorphic facial characteristics, brain structural malformations, developmental restriction, growth restriction, and neurocognitive disorders. The two major classes of chromosome 17p13.3 microduplication, which have different clinical presentations, are associated with specific genetic regions. Among the various known phenotypes, scattered cases with congenital heart disease (CHD) have been reported for both classes of chromosome 17p13.3 microduplication syndrome. Unfortunately, there is insufficient understanding of the correlation between chromosome anomaly induced alterations in gene expression and aberrant cardiac development, and thus early diagnosis of CHD among patients with chromosome 17p13.3 microduplication is difficult without routine prenatal cardiac assessment. One such congenital heart anomalies known to affect a substantial number of newborns worldwide is ventricular septal defect (VSD), which has been found in 17p13.3 microduplication carriers, and seems to sometimes undergo spontaneous closure. We report an unprecedented case of moderate sized perimembranous-outlet VSD and congestive heart failure (CHF) in a Chinese Han male infant with a class II chromosome 17p13.3 microduplication. Despite the fact that cytogenic testing and fetal echocardiography confirmed a 249-Kb chromosome duplication within 17p13.3 that encompassed the *PAFAH1B1* gene and showed the presence of VSD during prenatal period, this patient still developed a range of symptoms including sustained prolonged feeding, dyspnea, diaphoresis and retarded growth. A physical examination indicated hepatomegaly and a grade III/VI pan-systolic murmur along the left upper sternal border. Laboratory testing showed a high serum pro–B-type natriuretic peptide (pro-BNP). Imaging studies revealed cardiomegaly and a persistent VSD with related pulmonary stenosis. Since the clinical findings were compatible with CHF, we provided mainline treatment with digoxin, captopril, and furosemide, as well as fluid restriction. Despite sustained poor weight gain, the feeding behavior and the respiratory conditions of the patient improved gradually. This case report and literature review suggest that patients carrying chromosome 17p13.3 microduplication who have VSD may have an increased risk of developing CHF as young infants and hence a comprehensive cardiac evaluation is warranted to allow the early diagnosis and management of any severe heart anomalies.

## Introduction

17p13.3 microduplication syndrome, which seem to be created by non-allelic homologous recombination, is a rare genetic disorder and only 40 cases have been documented since it was first reported in 2009 (1, 2). Microduplications affecting 17p13.3 are categorized into two classes according to the affected genes within the duplication (3). In contrast to the absolute absence of *PAFAH1B1* duplications in class I 17p13.3 microduplications, an amplified *PAFAH1B1* gene and the possible concurrent duplication of the *YWHAE* gene or the *CRK* gene within the 17p13.3 region have been found in class II 17p13.3 microduplications (3). Class I microduplications are associated with learning disabilities, autism, overgrowth tendency, facial dysmorphism, and developmental delays (1, 3, 4). Class II microduplications otherwise lead to mild brain malformations, hypotonia and developmental delays (1, 3, 4). Despite early identification of this rearrangement being possible using advanced cytogenetic analysis techniques such as microarray comparative genomic hybridization (aCGH) (5, 6), a comprehensive understanding of all the clinical presentations brought about by chromosome17p13.3 microduplication and their genotype-phenotype correlations are lacking. This has limited the development of effective treatments. CHD in patients carrying chromosome 17p13.3 microduplication is particularly under-recognized due to its sporadic nature and poor in-depth descriptions of relevant cardiovascular evaluation details (5, 7).

CHD is one of the most commonly diagnosed congenital disorder and detrimentally impacts the health of 0.8% of live births worldwide (8, 9). Among the various types of CHDs, VSD accounts for a large proportion of congenital heart malformations (up to 40%), and has been reported to have a prevalence of 5.3% among newborns based on color doppler echocardiography (10). It is well-established that many VSD patients are asymptomatic and that their cardiac defect spontaneously closes without long-term effects (10). Nevertheless, because pulmonary vascular resistance during the early neonatal period is diminished, infants with non-restrictive VSDs can develop CHF due to persistent excessive pulmonary blood flow via the left-to-right interventricular shunt (10). This can lead to mortality unless there is timely surgical intervention (11).

We report a 2-month-old Chinese male infant with *de novo* microduplication of 17p13.3 encompassing *PAFAH1B1* who presented with feeding difficulties and restricted growth. In addition to a pansystolic murmur and hepatomegaly, laboratory tests and an echocardiogram showed elevated pro-BNP and a moderate-sized perimembranous-outlet VSD with related pulmonary stenosis. Our patient demonstrated a quite severe cardiac defect complicated by CHF. This is the first such reported case in an Asian population. We present this case to raise clinical awareness and broaden our understanding of the severe heart anomalies that occur in patients carrying a chromosome 17 microduplication.

## Case Presentation

A 2-month-old male infant was admitted with frequent vomiting during feeding, diaphoresis and dyspnea with subcostal retractions for 2 weeks. The patient had been bottle-fed with formula milk per feed (120 ml) four times daily (total fluid 129 ml/kg/24 h), and had received all appropriate vaccinations. However, prolonged feeding (more than 1 h per meal), with dysphagia and intermittent vomiting, and a decreased urine output with reddish-orange staining on diapers, had been present for about 2 weeks. The second child of non-consanguineous healthy Han Chinese parents, he was born at full term by vaginal delivery (Apgar scores of 8 and 9 at 1 and 5 min, respectively). His birth weight was 3,140 gm (10th−50th percentile), his height was 47.5 cm (10th−50th percentile), and his occipital frontal circumference was 35.5 cm (>90th percentile).

**Figure 1:**
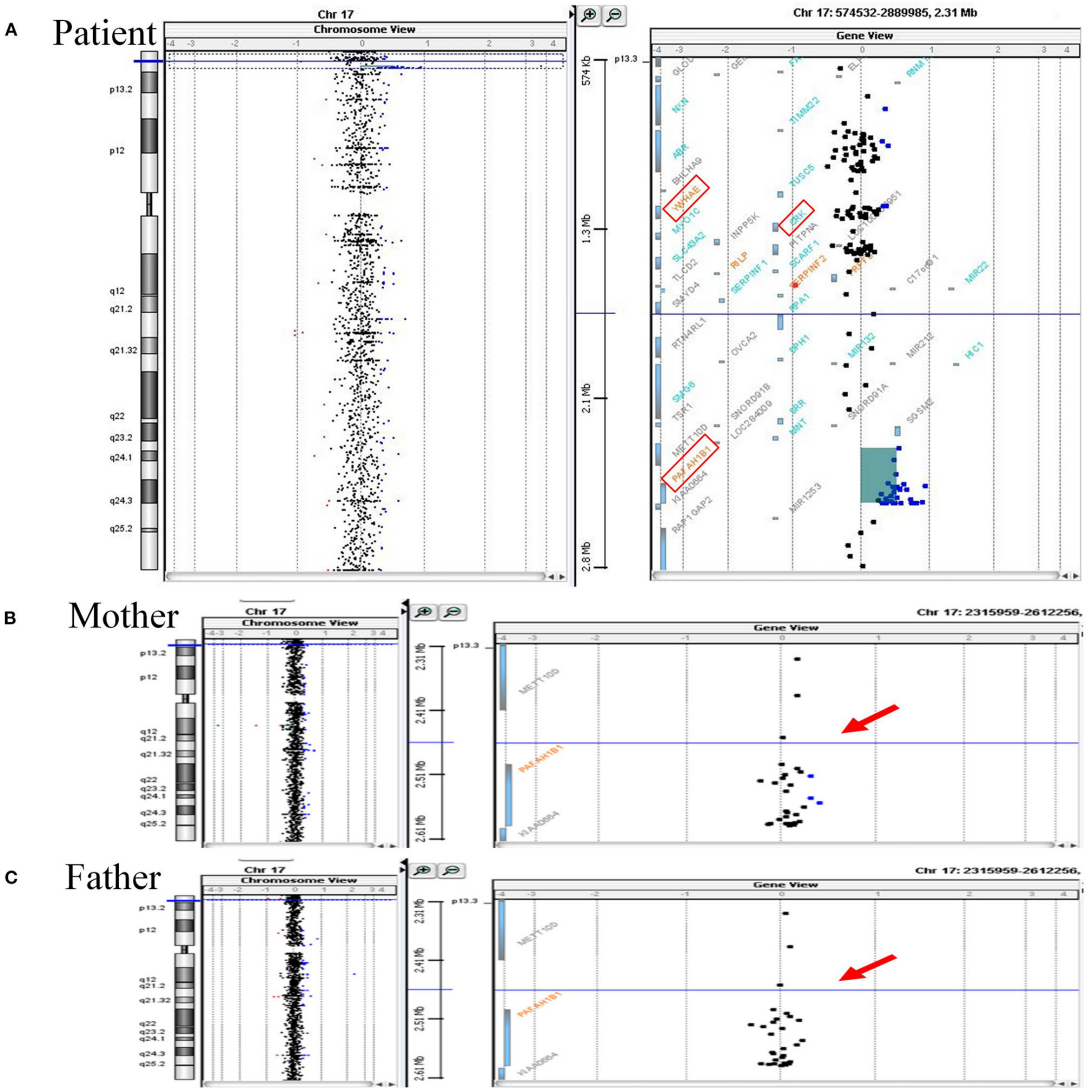
Array CGH analyses of chromosome 17 of the patient and his parents. **(A)** Array CGH detected a gain of 0.249 Mb (approximately log_2_ 1.5-fold increase) at chromosome 17p13.3 (2,339,561–2,588,655) in the patient. Increased relative fluorescence intensities are shown for a total of 29 labeled DNA probes (represented as the blue-colored dots). The duplicated chromosome region harbors *PAFAH1B1*, while another two candidate genes associated with chromosome 17p13.3 microduplication syndrome, namely *YWHAE* and *CRK*, have a normal copy number (the three candidate genes are marked by the red square frames). **(B,C)** The same genomic region (indicated by red arrows) of the patient's parents were essentially normal. Duplicated circular binary segmentation algorithms, consisting of the genomic position and the log_2_ copy number ratio, were plotted using Agilent CytoGenomics Software Version 1.5. Arabic numerals on the horizontal axis denote the log_2_ copy number ratio (from log_2_ ratios −4 to +4).

His mother, a 34-year-old multigravida, had been diagnosed with hyperthyroidism before pregnancy and had been treated with propylthiouracil 50 mg once daily. Both the father and the older sister of the patient were healthy. After being informed of a 1 in 61 chance of having a baby with Down syndrome based on her maternal serum screening, his mother had undergone amniocentesis at week 19 of gestation. The G-banded karyograms of the patient were normal (46 XY), however cytogenetic analysis of the fetal DNA by aCGH revealed a 0.249-Mb duplication on chromosome 17p13.3 [arr 17p13.3 (2,339,561–2,588,655) ×3] that encompassed *PAFAH1B1*, but not another candidate gene *YWHAE* (Figure 1A). Since oligonucleotide-based microarray analyses of both his parents gave normal profiles (Figures 1B,C), *de novo* chromosomal rearrangement was likely to be the cause of the chromosome 17 microduplication. Fetal MRI performed at week 28 of gestation revealed no significant abnormalities, whereas a fetal cardiac anomaly scan at week 34 of gestation found a 0.3-cm VSD.

The patient was born at 39 + 2 weeks of gestation via spontaneous vaginal delivery without fetal distress, and no obvious facial dysmorphism was documented in the newborn physical examination. After birth, due to shortness of breath with oxygen desaturation (SpO_2_ 85–90%) during feeding, the patient was admitted to neonatal intensive care for empirical antibiotic treatment under suspicion of neonatal sepsis. We discontinued antibiotics after two blood cultures separated by 48 h were negative. Cardiac sonography was performed and this detected a perimembranous-outlet type VSD. After discharge, digoxin therapy at 4.5 mcg/kg twice daily was initiated and the patient was regularly followed-up at an outpatient pediatric cardiology clinic.

**Figure 2:**
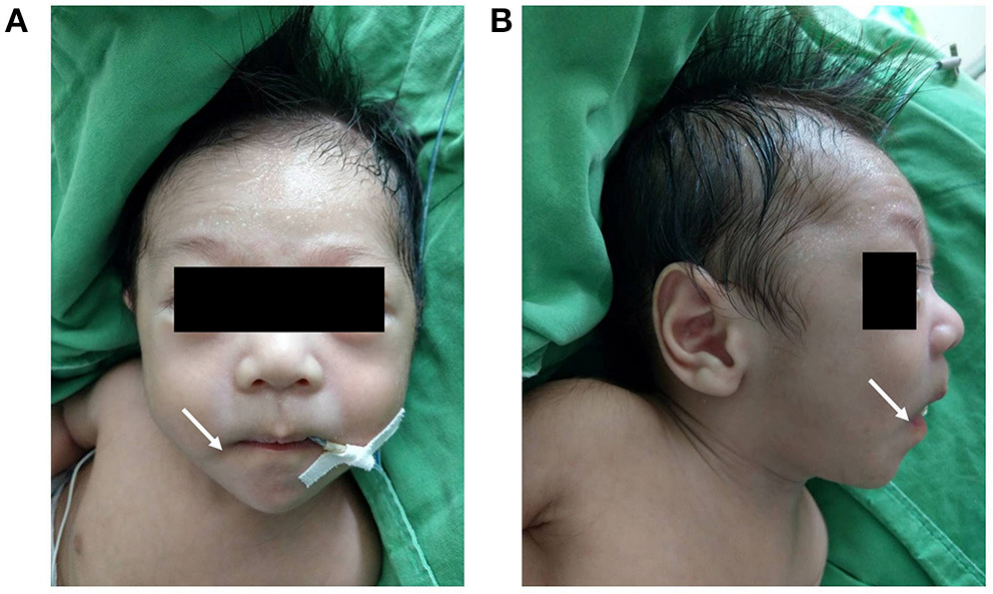
Anterior and lateral views of the patient's face **(A,B)** revealing dolichocephaly, a down-turned mouth (indicated by the white arrow), a triangular face and diaphoresis on the forehead.

The physical examination on admission showed a blood pressure of 86/47 mmHg, a respiratory rate of 49/min, a heart rate of 147/min, and a body temperature of 37.1°C. Notwithstanding, his body weight and height were below the 10th percentile of the same age group (body weight 3.71 kg, and height 54 cm). Moreover, the patient presented with dysmorphic facial features, including dolichocephaly, a down-turned mouth, and a triangular face (Figures 2A,B). Cardiac auscultation and abdomen palpitation revealed a grade III/VI pan-systolic murmur over left upper sternal border and hepatomegaly at 2.5 finger-breaths below the costal margin. Otherwise, no significant developmental delay or neurobehavioral abnormalities were found.

**Figure 3:**
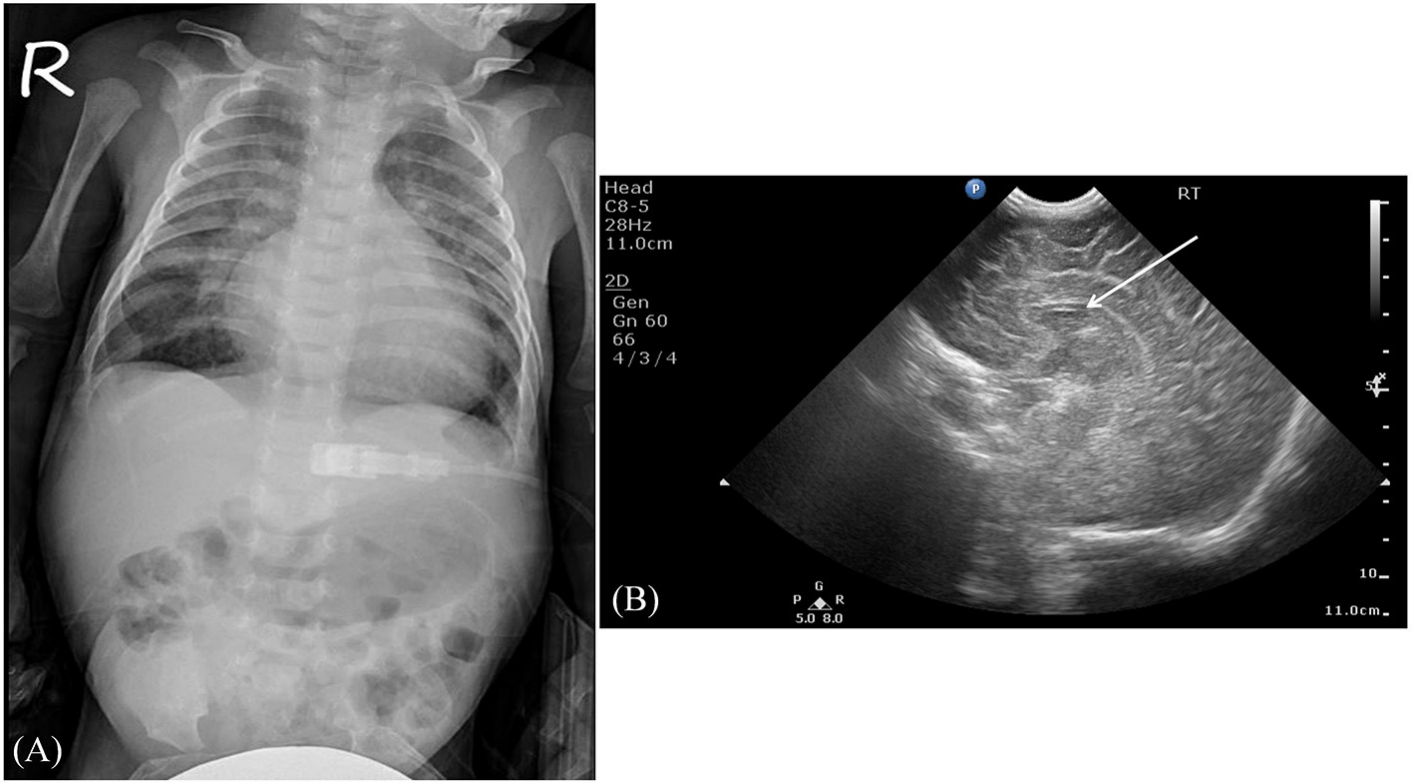
Imaging studies. **(A)** The chest radiography revealed cardiomegaly and pulmonary congestion. **(B)** A sagittal view using brain sonography showed a subtle hypoplasia of the corpus callosum (indicated by the white arrow).

**Figure 4:**
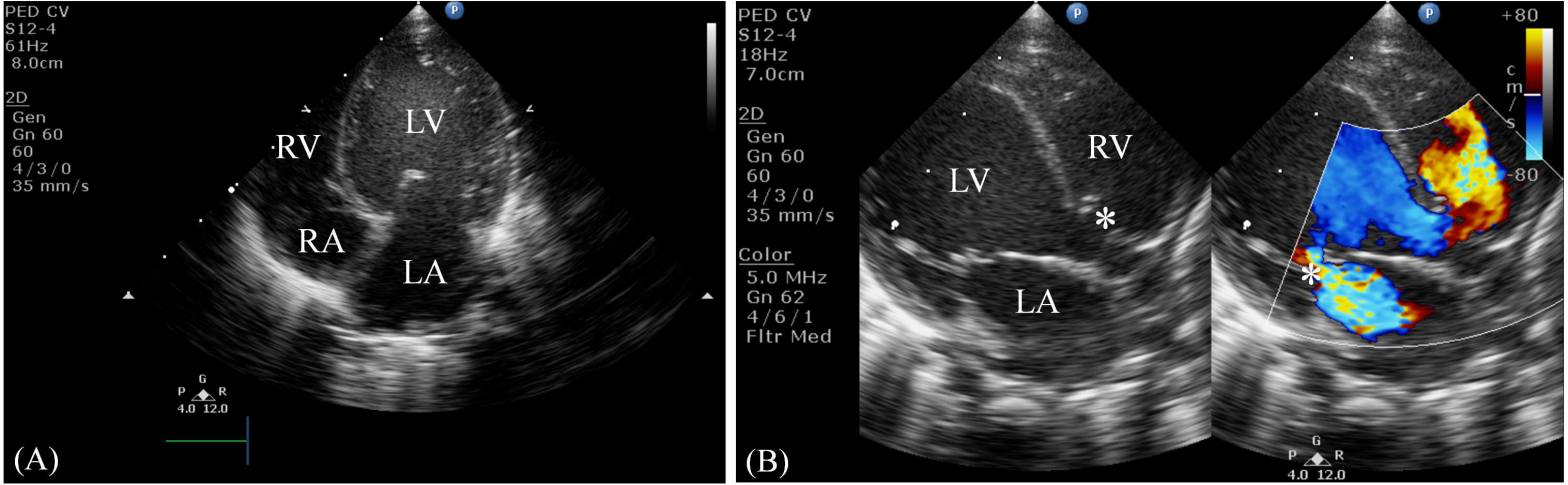
Two-dimensional echocardiogram. **(A)** This apical four-chamber view shows dilation of the left ventricle and atrium. **(B)** This parasternal long-axis view demonstrates a non-restrictive perimembranous-outlet type VSD sized 0.471 cm (indicated by the left white asterisk) and moderate mitral valve regurgitation (indicated by the right white asterisk). LV, left ventricle; RV, right ventricle; LA, left atrium; RA, right atrium.

Serum laboratory testing revealed elevated pro-BNP (3,119 pg/ml, reference range 5–1,121 pg/ml) without electrolyte imbalance. Chest radiography showed cardiomegaly (cardiothoracic ratio 0.66) and pulmonary congestion (Figure 3A). An echocardiogram on admission identified a perimembranous-outlet VSD (0.471 cm) with related pulmonary stenosis and a dilated left-sided heart with mild-to-moderate mitral valve regurgitation (Ejection Fraction 86.4%, and Left Atrial to Aortic Root Ratio 1.84) (Figures 4A,B). Brain malformations may exist in patients carrying chromosome 17p13.3 microduplications, and therefore cranial sonography was performed. This showed subtle corpus callosum hypoplasia (Figure 3B). Furthermore, abdomen sonography excluded infantile hypertrophic pyloric stenosis, a likely cause of an infant presenting with frequent vomiting, lethargy and body weight loss.

To strike a balance between fluid overload control and adequate nutrition supplementation, we gradually titrated the amount of formula milk fed to the patient (30 kcal/ounce) until his daily caloric intake reached 150 kcal/kg/day, while at the same time the daily total fluid amount given to the patient was limited, to not exceeding 150 mg/kg/day. An oral form loop diuretic, furosemide (2 mg/kg/dose), was given to the patient twice daily in order to maintain an adequate urine output of 2 ml/kg/hr. Heart failure medications, including digoxin (4 mcg/kg/dose) and captopril (0.2 mg/kg/dose), were given to the infant twice daily. In addition, we used domperidone (0.25 mg/kg/dose) every 8 h to provide relief from vomiting and reduced difficulty with feeding. During admission, plasma levels of pro-BNP and electrolytes, as well as chest radiography images, were obtained regularly to evaluate the efficacy of the anticongestive treatment. Despite sustained perihilar infiltrates and an increased cardiothoracic ratio, as shown in chest x-ray images, the heart failure symptoms improved gradually and were accompanied by clinical evidence of an alleviation of the patient's dyspnea. There was also a gradual improvement in both oral intake and urine output, together with a steadily decrease in plasma pro-BNP and an absence of electrolyte imbalance. Repeated echocardiograms after treatment revealed that the size of the VSD was unchanged at 0.471 cm, and this was accompanied by a subtle decrease in the left atrial to aortic root ratio (Ejection Fraction 85%, LA/Ao 1.5). An intramuscular palivizumab injection was given once per month to prevent respiratory syncytial virus infection. Developmental delay among patients carrying a chromosome 17p13.3 microduplication is common and therefore we consulted a pediatric neurologist. No marked abnormalities were noted after stringent evaluation.

The patient was discharged from the hospital given the refusal of surgical intervention by his mother. Nevertheless, his mother changed her mind shortly after, and therefore the infant underwent surgical closure of the VSD at another medical center in Northern Taiwan. According to his mother, the infant was in a stable condition after the operation. He is 7-month-old now and is regularly followed up at the pediatric cardiology/neurology outpatient department without any report of development delay.

## Discussion

Among a wide spectrum of phenotypes, congenital heart disease is rare for both classes of chromosome 17p13.3 microduplication syndrome (5). Specifically, reports have identified patent ductus arterious, mitral valve prolapse, aortic dilation and aortic stenosis as common among class I 17p13.3 microduplication carriers, while patient foramen ovale and spontaneously closing VSD have been identified among class II individuals (4, 5, 7, 12). Cytogenetic testing of probands and their parents has proved that these gene amplifications usually originate by *de novo* chromosomal rearrangement, including ours. In our case the amplification is a plausible cause of this patient's CHD (2). Notably, distinct from spontaneously closing VSD, which was asymptomatic in the case described by Avela et al. (7), it is unprecedented that our patient, with the smallest duplicated chromosome fragment of all existing cases, should suffer from a non-restrictive VSD and then subsequently develop CHF.

Distinct from the signs and symptoms of heart failure in adults, infants with CHF suffer from fatigue, difficulty in feeding, shortness of breath, diaphoresis, retarded growth and retarded development (13–15). Physical findings may include tachypnea or dyspnea with grunting and retractions, tachycardia, a gallop rhythm and hepatomegaly (13, 16). Electrocardiogram and chest X-ray images can exclude cardiac arrhythmia and provide evidence supporting CHF, including cardiomegaly (cardiothoracic ratio > 0.6 cm on a posterior anterior film), pulmonary edema and pleural effusion (14, 17). B-type natriuretic peptide (BNP) and N-terminal pro BNP assays are widely used and have the clinical benefit of excluding non-cardiac causes of respiratory symptoms and fluid retention, of assessing treatment efficacy, of predicting future hospitalization and of pinpointing mortality risk (18–20). Cardiac troponin I and T are also useful biomarkers that can reflect myocyte injury, but they are less specific to heart failure because elevated cardiac enzyme levels are often present in other clinical scenarios including myocarditis or ischemic heart disease (13, 14). Monitoring of electrolyte levels, liver function and renal function are crucial for identifying impairment of vital organs due to poor perfusion; they and can serve as indicators during diuretic dose adjustment (14). Additionally, a complete blood count and thyroid function testing may reveal the presence of aggravating factors for heart failure, including anemia, hyperthyroidism or hypothyroidism (14). Both the clinical symptoms and objective findings of our patient were consistent with CHF; it should be noted that electrolyte imbalance and/or vital organ impairment were not present on admission.

Neonates with CHDs, including VSDs, complete atrioventricular canal defects, patent ductus arteriosus and aortoepulmonary windows, are susceptible to develop CHF when a persistent left to right shunt and increased pulmonary blood flow due to reduced pulmonary resistance are present (10, 14). Nevertheless, the correlation between chromosomal duplication and cardiac structural abnormalities remains unclear given the low number of confirmed cases. Based on various studies, involving animal models, we suggest that alterations in the expression of *YWHAE* and *PAFAH1B* may result in a genetic predisposition toward heart failure among 17p13.3 microduplications carriers (21–26).

Both trabeculation and compaction are essential processes during cardiogenesis (27). The trabecular myocardium makes up the majority of the embryonic ventricles initially, and a gradual replacement of the myocardial trabeculum by a compact myocardium occurs during the later stages of heart development (21, 27, 28). Mutation of genes regulating ventricular growth and maturation seem to lead to CHD and even heart failure (28). Previous studies have shown that *YWHAE* encodes 14-3-3ε, a protein that specifically binds to ubiquitous phosphoserine/threonines and is crucial for promoting human endothelial cell survival and regulating ventricular myocardium compaction. It has been shown to do this in a mouse model by modulating the cardiomyocyte cell cycle (21, 22). In Kosaka et al., which explored heart development in mice carrying a homozygous 14-3-3ε deletion, similar histological characteristics to left ventricular non-compaction cardiomyopathy in humans were found. Furthermore, it was shown that defective 14-3-3ε functioning is detrimental to interventricular septum maturation resulting in concomitant VSDs in ~60% of 14-3-3ε-null mice (21). Despite an unclear role for the 14-3-3ε protein in ventricular morphogenesis, the presence of cell cycle arrest, was detectable. This was associated with upregulation of the G1/S phase transition inhibitor p27Kip1 and downregulation of cyclin E1 in the 14-3-3ε-deficient cardiomyocytes, providing a tentative explanation for the phenotype (21).

*PAFAH1B1* is another candidate gene that has been postulated to impact cardiovascular maturation and this seems to occur via regulation of cardiac-specific microRNAs. The protein encoded by *PAFAH1B1*, platelet-activating factor acetylhydrolase IB subunit alpha, ensures appropriate cell migration by stabilizing dynein-dependent microtubule dynamics (29). Rao et al. demonstrated that cardiac-specific deletion of the miRNA-processing enzyme Dicer results in dilated cardiomyopathy and premature death (23). Furthermore, microRNA-125b, a cardiomyocyte maturation inducer, seems to protect murine cardiac myocytes against myocardium ischemia/reperfusion injury by suppressing the pro-apoptotic genes *p53* and *Bak1* (24). Moreover, defective mitochondrial morphology and dysregulation of fatty acid metabolism were found to be present in the hearts of miR-125b-1 knockout mice resulting in cardiac hypertrophy and high prenatal lethality (25). Intriguingly, in addition to the identification of *PAFAH1B1* as a target of miR-139-5p by proteomics analysis, the expression of Pafah1b1 protein is known to be upregulated after inhibition of microRNA-125b (25, 26). While the direct influence of Pafah1b1 protein on cardiovascular development remains open, the aforementioned findings imply that it potentially can hamper normal cardiac morphogenesis. The aCGH analysis carried out on our patient only showed the presence of a *PAFAH1B1* amplification and confirmed that the *YWHAE* gene had a normal copy number. We hypothesize that the CHF symptoms, which originated from non-restricted VSD, may be aggravated by 17p13 microduplications encompassing *PAFAH1B1*. This seems to result in an increased susceptibility to cardiac hypertrophy and thus compromised cardiac energy production.

Apart from influencing cardiomyocyte maturation, increased expression of *PAFAH1B1* and its protein LIS1 have been shown to induce subtle brain malformations, to retard growth and to cause moderate/severe developmental delay (4). Among the documented brain abnormalities in patients with class II 17p13.3 microduplication syndrome, a significant number of cases have a hypoplastic/dysplastic corpus callosum, a smaller occipital cortex and a mild cerebellum volume loss (1, 4). Mild corpus callosum hypoplasia was detected in our patient by cranial ultrasound imaging rather than more widely used brain MRI (1, 4, 7, 12), regular neurological assessments of similar cases is suggested.

The facial characteristics present in this case are somewhat similar to the ones described in another patient with class II 17p13.3 microduplication (7). Nevertheless, it remains challenging when trying to differentiate the two classes of 17p13.3 microduplication syndrome based on dysmorphic facial features because there are several unsolved problems. Firstly, subtle facial dysmorphism tends to be misrecognized/neglected when an experienced clinical geneticist is not available; therefore whether existing reports describe accurately the facial characteristics of each patient is uncertain. Secondly, despite the fact that prior studies have shown a strong correlation between dysmorphic facial traits and changes affecting the *YWHAE*/*CRK* genes (3, 5, 30, 31), evidence confirming the genetic impact of *PAFAH1B1* amplification and a normal *YWHAE* genotype on the facial development of patients remains lacking. Further investigations are required to decipher the causative factor(s) behind facial dysmorphism in patients with chromosome 17p13.3 microduplication.

In conclusion, we describe for the first time a case involving a Chinese Han male infant with a class II chromosome 17p13.3 microduplication who developed non-restrictive VSD and significant CHF. In addition to the previously identified phenotypes associated with chromosome 17p13.3 microduplication, including brain structural anomalies and facial dysmorphism, pediatricians should be aware of the possibility of severe heart structural defects and related complications when patients carry 17p13.3 microduplications. Moreover, the present case highlights the role of aCGH in detecting chromosomal anomalies in fetuses with CHD, for those patients have been shown to carry a higher risk of additional extracardiac malformations (32). Compared with conventional karyotyping, high resolution aCGH enables more accurate detection of copy number variants within the genome (32). The case report also echoes the comments made by Ho et al., who emphasized the importance of comprehensive non-invasive cardiac evaluations when patients have a 17p13.3 microduplication (5). There has been increased application of early diagnosis by prenatal cytogenetic analysis and fetal cardiac ultrasound scanning is now more common. This meant that our patient received timely treatment and did not undergo progressive deterioration of his heart and possible heart failure. This case report broadens our understanding of congenital heart disease among chromosome 17p13.3 microduplication carriers, while at the same time emphasizing the need for additional research to unravel the underlying pathogenesis associated with chromosome 17p13.3 microduplications.

## Data Availability Statement

The original contributions presented in the study are included in the article/supplementary material, further inquiries can be directed to the corresponding author/s.

## Ethics Statement

The studies involving human participants were reviewed and approved by Tri-Service General Hospital. Written informed consent to participate in this study was provided by the participants' legal guardian/next of kin. Written informed consent was obtained from the minor(s)' legal guardian/next of kin for the publication of any potentially identifiable images or data included in this article.

## Author Contributions

Y-YY collected the data, analyzed the data, and wrote the manuscript. C-TL and L-FP participated in the patient's clinical care and collected the data. C-FH aided in interpreting the results. S-JC revised the manuscript. W-FH participated in the patient's clinical care and contributed to the final version of the manuscript. All authors contributed to the article and approved the submitted version.

## Conflict of Interest

The authors declare that the research was conducted in the absence of any commercial or financial relationships that could be construed as a potential conflict of interest.

## Publisher's Note

All claims expressed in this article are solely those of the authors and do not necessarily represent those of their affiliated organizations, or those of the publisher, the editors and the reviewers. Any product that may be evaluated in this article, or claim that may be made by its manufacturer, is not guaranteed or endorsed by the publisher.

## References

[1] Curry CJ Rosenfeld JA Grant E Gripp KW Anderson C Aylsworth AS. The duplication 17p13.3 phenotype: analysis of 21 families delineates developmental, behavioral and brain abnormalities, and rare variant phenotypes. Am J Med Genet A. (2013) 161A:1833–52. 10.1002/ajmg.a.35996 23813913 PMC5517092

[2] Blazejewski SM Bennison SA Smith TH Toyo-Oka K. Neurodevelopmental genetic diseases associated with microdeletions and microduplications of chromosome 17p13.3. Front Genet. (2018) 9:80. 10.3389/fgene.2018.00080 29628935 PMC5876250

[3] Bruno DL Anderlid BM Lindstrand A van Ravenswaaij-Arts C Ganesamoorthy D Lundin J. Further molecular and clinical delineation of co-locating 17p13.3 microdeletions and microduplications that show distinctive phenotypes. J Med Genet. (2010) 47:299–311. 10.1136/jmg.2009.069906 20452996

[4] Bi W Sapir T Shchelochkov OA Zhang F Withers MA Hunter JV. Increased LIS1 expression affects human and mouse brain development. Nat Genet. (2009) 41:168–77. 10.1038/ng.302 19136950 PMC4396744

[5] Ho AC Liu AP Lun KS Tang WF Chan KY Lau EY. A newborn with a 790 kb chromosome 17p13.3 microduplication presenting with aortic stenosis, microcephaly and dysmorphic facial features - is cardiac assessment necessary for all patients with 17p13.3 microduplication? Eur J Med Genet. (2012) 55:758–62. 10.1016/j.ejmg.2012.09.011 23063769

[6] Chen CP Chang TY Guo WY Wu PC Wang LK Chern SR. Chromosome 17p13.3 deletion syndrome: aCGH characterization, prenatal findings and diagnosis, and literature review. Gene. (2013) 532:152–9. 10.1016/j.gene.2013.09.044 24055730

[7] Avela K Aktan-Collan K Horelli-Kuitunen N Knuutila S Somer M. A microduplication on chromosome 17p13.1p13.3 including the PAFAH1B1 (LIS1) gene. Am J Med Genet A. (2011) 155A:875–9. 10.1002/ajmg.a.33944 21595003

[8] Bernier PL Stefanescu A Samoukovic G Tchervenkov CI. The challenge of congenital heart disease worldwide: epidemiologic and demographic facts. Semin Thorac Cardiovasc Surg Pediatr Card Surg Annu. (2010) 13:26–34. 10.1053/j.pcsu.2010.02.005 20307858

[9] Bouma BJ Mulder BJ. Changing landscape of congenital heart disease. Circ Res. (2017) 120:908–22. 10.1161/CIRCRESAHA.116.309302 28302739

[10] Penny DJ Vick GW III. Ventricular septal defect. Lancet. (2011) 377:1103–12. 10.1016/S0140-6736(10)61339-6 21349577

[11] Sommers C Nagel BH Neudorf U Schmaltz AA. [Congestive heart failure in childhood. An epidemiologic study]. Herz. (2005) 30:652–62. 10.1007/s00059-005-2596-6 16333593

[12] Roos L Jonch AE Kjaergaard S Taudorf K Simonsen H Hamborg-Petersen B. A new microduplication syndrome encompassing the region of the Miller-Dieker (17p13 deletion) syndrome. J Med Genet. (2009) 46:703–10. 10.1136/jmg.2008.065094 19520700

[13] Rossano JW Shaddy RE. Heart failure in children: etiology and treatment. J Pediatr. (2014) 165:228–33. 10.1016/j.jpeds.2014.04.055 24928699

[14] Masarone D Valente F Rubino M Vastarella R Gravino R Rea A. Pediatric heart failure: a practical guide to diagnosis and management. Pediatr Neonatol. (2017) 58:303–12. 10.1016/j.pedneo.2017.01.001 28279666

[15] Nandi D Rossano JW. Epidemiology and cost of heart failure in children. Cardiol Young. (2015) 25:1460–8. 10.1017/S1047951115002280 26675591 PMC4763311

[16] Madriago E Silberbach M. Heart failure in infants and children. Pediatr Rev. (2010) 31:4–12. 10.1542/pir.31.1.4 20048034

[17] Satou GM Lacro RV Chung T Gauvreau K Jenkins KJ. Heart size on chest x-ray as a predictor of cardiac enlargement by echocardiography in children. Pediatr Cardiol. (2001) 22:218–22. 10.1007/s002460010207 11343146

[18] Price JF Thomas AK Grenier M Eidem BW O'Brian Smith E Denfield SW. B-type natriuretic peptide predicts adverse cardiovascular events in pediatric outpatients with chronic left ventricular systolic dysfunction. Circulation. (2006) 114:1063–9. 10.1161/CIRCULATIONAHA.105.608869 16940194

[19] Auerbach SR Richmond ME Lamour JM Blume ED Addonizio LJ Shaddy RE. BNP levels predict outcome in pediatric heart failure patients: *post hoc* analysis of the Pediatric Carvedilol Trial. Circ Heart Fail. (2010) 3:606–11. 10.1161/CIRCHEARTFAILURE.109.906875 20573993

[20] Wong DT George K Wilson J Manlhiot C McCrindle BW Adeli K. Effectiveness of serial increases in amino-terminal pro-B-type natriuretic peptide levels to indicate the need for mechanical circulatory support in children with acute decompensated heart failure. Am J Cardiol. (2011) 107:573–8. 10.1016/j.amjcard.2010.10.015 21295174

[21] Kosaka Y Cieslik KA Li L Lezin G Maguire CT Saijoh Y. 14-3-3epsilon plays a role in cardiac ventricular compaction by regulating the cardiomyocyte cell cycle. Mol Cell Biol. (2012) 32:5089–102. 10.1128/MCB.00829-12 23071090 PMC3510533

[22] Brunelli L Cieslik KA Alcorn JL Vatta M Baldini A. Peroxisome proliferator-activated receptor-delta upregulates 14-3-3 epsilon in human endothelial cells via CCAAT/enhancer binding protein-beta. Circ Res. (2007) 100:e59–71. 10.1161/01.RES.0000260805.99076.22 17303761

[23] Rao PK Toyama Y Chiang HR Gupta S Bauer M Medvid R. Loss of cardiac microRNA-mediated regulation leads to dilated cardiomyopathy and heart failure. Circ Res. (2009) 105:585–94. 10.1161/CIRCRESAHA.109.200451 19679836 PMC2828903

[24] Wang X Ha T Zou J Ren D Liu L Zhang X. MicroRNA-125b protects against myocardial ischaemia/reperfusion injury via targeting p53-mediated apoptotic signalling and TRAF6. Cardiovasc Res. (2014) 102:385–95. 10.1093/cvr/cvu044 24576954 PMC4030511

[25] Chen CY Lee DS Choong OK Chang SK Hsu T Nicholson MW. Cardiac-specific microRNA-125b deficiency induces perinatal death and cardiac hypertrophy. Sci Rep. (2021) 11:2377. 10.1038/s41598-021-81700-y 33504864 PMC7840921

[26] Oh JG Lee P Gordon RE Sahoo S Kho C Jeong D. Analysis of extracellular vesicle miRNA profiles in heart failure. J Cell Mol Med. (2020) 24:7214–27. 10.1111/jcmm.15251 32485073 PMC7339231

[27] Wengrofsky P Armenia C Oleszak F Kupferstein E Rednam C Mitre CA. Left ventricular trabeculation and noncompaction cardiomyopathy: a review. EC Clin Exp Anat. (2019) 2:267–83.31799511

[28] Wilsbacher L McNally EM. Genetics of cardiac developmental disorders: cardiomyocyte proliferation and growth and relevance to heart failure. Annu Rev Pathol. (2016) 11:395–419. 10.1146/annurev-pathol-012615-044336 26925501 PMC8978617

[29] Abal M Piel M Bouckson-Castaing V Mogensen M Sibarita JB Bornens M. Microtubule release from the centrosome in migrating cells. J Cell Biol. (2002) 159:731–7. 10.1083/jcb.200207076 12473683 PMC2173398

[30] Ostergaard JR Graakjaer J Brandt C Birkebaek NH. Further delineation of 17p13.3 microdeletion involving CRK. The effect of growth hormone treatment. Eur J Med Genet. (2012) 55:22–6. 10.1016/j.ejmg.2011.09.004 22085993

[31] Barros Fontes MI Dos Santos AP Rossi Torres F Lopes-Cendes I Cendes F Appenzeller S. 17p13.3 Microdeletion: insights on genotype-phenotype correlation. Mol Syndromol. (2017) 8:36–41. 10.1159/000452753 28232781 PMC5260540

[32] Jansen F Blumenfeld Y Fisher A Cobben J Odibo A Borrell A. Array comparative genomic hybridization and fetal congenital heart defects: a systematic review and meta-analysis. Ultrasound Obstet Gynecol. (2015) 45:27–35. 10.1002/uog.14695 25319878

